# Gut microbiome alterations in immune thrombocytopenia: a systematic review of current evidence

**DOI:** 10.3389/fmed.2025.1511612

**Published:** 2025-05-16

**Authors:** Alireza Khiabani, Roohollah Mirzaee Khalilabadi, Hajar Mardani Valandani, Zahra Khoshnegah, Alireza Khanahmad, Hojat Shahraki, Najmeh Nezamabadipour, Alireza Farsinejad, Mehran Rahimlou

**Affiliations:** ^1^Student Research Committee, Faculty of Allied Medicine, Kerman University of Medical Sciences, Kerman, Iran; ^2^Department of Hematology and Medical Laboratory Sciences, Faculty of Allied Medicine, Kerman University of Medical Sciences, Kerman, Iran; ^3^Department of Hematology and Blood Banking, School of Allied Medical Sciences, Shahid Beheshti University of Medical Sciences, Tehran, Iran; ^4^School of Medicine, Bam University of Medical Sciences, Bam, Iran; ^5^Stem Cells and Regenerative Medicine Innovation Center, Kerman University of Medical Sciences, Kerman, Iran; ^6^Metabolic Diseases Research Center, Health and Metabolic Research Institute, Zanjan University of Medical Science, Zanjan, Iran

**Keywords:** immune thrombocytopenia, gut microbiome, dysbiosis, autoimmune diseases, microbial diversity

## Abstract

**Background:**

Immune thrombocytopenia (ITP) is an autoimmune disorder characterized by immune-mediated platelet destruction and impaired platelet production. Recent evidence suggests a role for gut microbiome dysbiosis in autoimmune diseases, but its association with ITP remains unclear. This systematic review explores the potential link between the gut microbiome and ITP pathophysiology.

**Methods:**

We conducted a comprehensive search in five databases (MEDLINE, Scopus, Web of Science, Cochrane Library, Embase) from 1980 to July 2024, adhering to PRISMA 2020 guidelines. Studies assessing the gut microbiome in patients with ITP were included. The primary outcome was alterations in gut microbiota composition, and study selection was performed in three phases, with discrepancies resolved through consensus.

**Results:**

From 480 studies screened, 12 met the inclusion criteria. The studies revealed significant alterations in gut microbiota composition, particularly at the phylum level. An increase in Bacteroidetes and Proteobacteria was observed in some studies, while others reported a decrease in these phyla. Firmicutes showed inconsistent results across studies. Alpha and beta diversity analysis also yielded conflicting results, with some studies reporting decreased diversity, while others found no significant difference or an increase.

**Conclusion:**

The results suggest a potential link between gut microbiota dysbiosis and ITP, though findings remain inconsistent across studies. Further well-designed research is needed to clarify the role of the microbiome in ITP, with implications for novel therapeutic approaches.

## Introduction

Immune thrombocytopenia (ITP), formerly known as Idiopathic thrombocytopenic purpura or Immune thrombocytopenic purpura, is an important cause of acquired thrombocytopenia worldwide (1). ITP can cause Immune-mediated destruction of platelets, dysfunction of megakaryocytes, and platelet production defects through humoral (2) or cellular (3) immunity (4). No other clinical findings are seen except for bleeding and the final diagnosis is reached after ruling out other causes of thrombocytopenia (5). Research examining immune cells in individuals with ITP has revealed variations in the proportion and behavior of specific T cells, B cells, and plasmacytoid dendritic cells (pDCs) when compared to individuals without the condition (6–8). At the outset, specialized immune cells known as antigen-presenting cells (APCs), including macrophages, dendritic cells, and B cells, activate T cells with an autoimmune response. These autoreactive T cells, in conjunction with T follicular helper cells (Tfh), subsequently encourage the growth and development of autoreactive B cells into plasma cells, which then generate antibodies that target platelets (8).

According to the latest recommendations from the American Society of Hematology (ASH) and the International Working Group (IWG), treatment for ITP should primarily be guided by the presence of bleeding rather than platelet count alone. However, treatment is generally recommended for patients with platelet counts below 10–30 × 10^9^/L or those at high risk of bleeding. First-line therapy includes corticosteroids (e.g., prednisone, dexamethasone) and intravenous immunoglobulin (IVIg) for acute cases. Thrombopoietin receptor agonists (TPO-RAs), rituximab, and fostamatinib are now the preferred second-line therapies, while splenectomy is typically reserved for later treatment stages. Other immunosuppressive agents such as azathioprine and danazol may be considered in refractory cases but are supported by less robust evidence (9).

Several studies show the high prevalence of dysbiosis in patients with autoimmune diseases (10). The human intestine is home to trillions of enteric microbes, which constitute the most intricate part of the immune system. These microorganisms play crucial roles in both healthy bodily functions and the development of diseases (11). Intestinal microbiome imbalance plays a significant role in the development of various diseases. Recent studies have shown the role of the microbiome in prevention, treatment, and prognosis of malignancies (12, 13). Previous studies also clarified the relationship between the microbiome and several non-malignant diseases, such as cardiac (14, 15) or metabolic diseases (16–18) as well as a variety of extra-intestinal autoimmune diseases, including multiple sclerosis (MS), rheumatoid arthritis, type 1 diabetes, and systemic lupus erythematosus (19). However, the results of studies regarding the relationship between the microbiome and ITP have been very heterogeneous and somehow contradictory.

This systematic review aims to establish an association between the microbiome and the pathophysiology of ITP. This study also provides new insights into the limitations of previous literature to discuss the possible causes of discrepancies.

## Methods

The research methodology adhered to the systematic review guidelines outlined in the Cochrane Handbook (20) and presented its findings in accordance with the Preferred Reporting Items for Systematic Reviews and Meta-Analyses (PRISMA) 2020 statement (21).

### Information sources and search strategy

We performed an exhaustive search in MEDLINE via PubMed, Scopus, Web of Science, the Cochrane library and Embase since 1980 until July 2024. No restrictions to language or publication status were applied. Proper Boolean operators and database filters were applied to optimize the search. Before implementation, the research strategies were evaluated by another author (MR) using the Peer Review of Electronic Search Strategies Checklist (22). AKH conducted the literature search, while MR assessed the results. The reference sections of relevant reviews and included studies were examined to identify additional sources. To capture a comprehensive picture of current research, searches were conducted in July 2024 using Google Scholar and ClinicalTrials.gov to identify any ongoing or unpublished studies not yet reflected in published literature. Additionally, experts in the field were contacted to inquire about any ongoing studies not yet registered in public databases.

### Eligibility criteria

To ensure a rigorous systematic review, we have refined our eligibility criteria to clearly define the population, comparator, and study designs included in our analysis. The revised criteria are as follows:

**Population**: Studies investigating patients diagnosed with primary or secondary ITP, regardless of age, sex, or disease severity. ITP was defined according to standardized diagnostic criteria, including those from ASH and IWG.

**Comparator**: Studies comparing gut microbiota composition in ITP patients to healthy controls or other disease populations were included to assess microbial differences.

**Study design**: Eligible studies included case-control, cohort, cross-sectional, and interventional studies that analyzed gut microbiota using sequencing-based techniques (e.g., 16S rRNA sequencing, metagenomics, or metabolomics).

**Exclusion criteria**: Studies that lacked full-text availability, did not report original microbiota data, or focused solely on non-human models were excluded.

A three-phase approach was employed to choose studies, with identified references independently reviewed for inclusion by two authors (NN and AF). The initial stage involved examining titles obtained through the aforementioned systematic searches. Articles were included in the first screening if the title or abstracts mentioned microbiome and/or ITP. Subsequently, we reviewed the abstracts of all potentially relevant articles. Full-text articles fulfilling the criteria were independently chosen and examined by both authors to determine their suitability for the study. Any discrepancies between the two authors were addressed through discussion or by seeking input from another author (HMV). If multiple publications (like posters or peer-reviewed journal articles) existed, we selected only the published paper.

### Data extraction

Independent data extraction on the characteristics of the eligible studies was carried out by pairs of authors (AKH and MR, NN and AF). A pre-constructed Microsoft Excel 365 spreadsheet (Microsoft, Redmond, WA, USA) was employed in data extraction processes. The extracted data was reviewed for errors by a third author (HSH). The risk of bias in each included study was independently assessed by two authors (MR and NN). If there were any discrepancies, they were discussed between the two authors, and a third author (AKH) was consulted for a final assessment.

### Data synthesis

Due to the high methodological heterogeneity between the included studies, meta-analysis was not possible in this study, and the study was designed as a systematic review.

## Results

### Study characteristics

**Figure 1:**
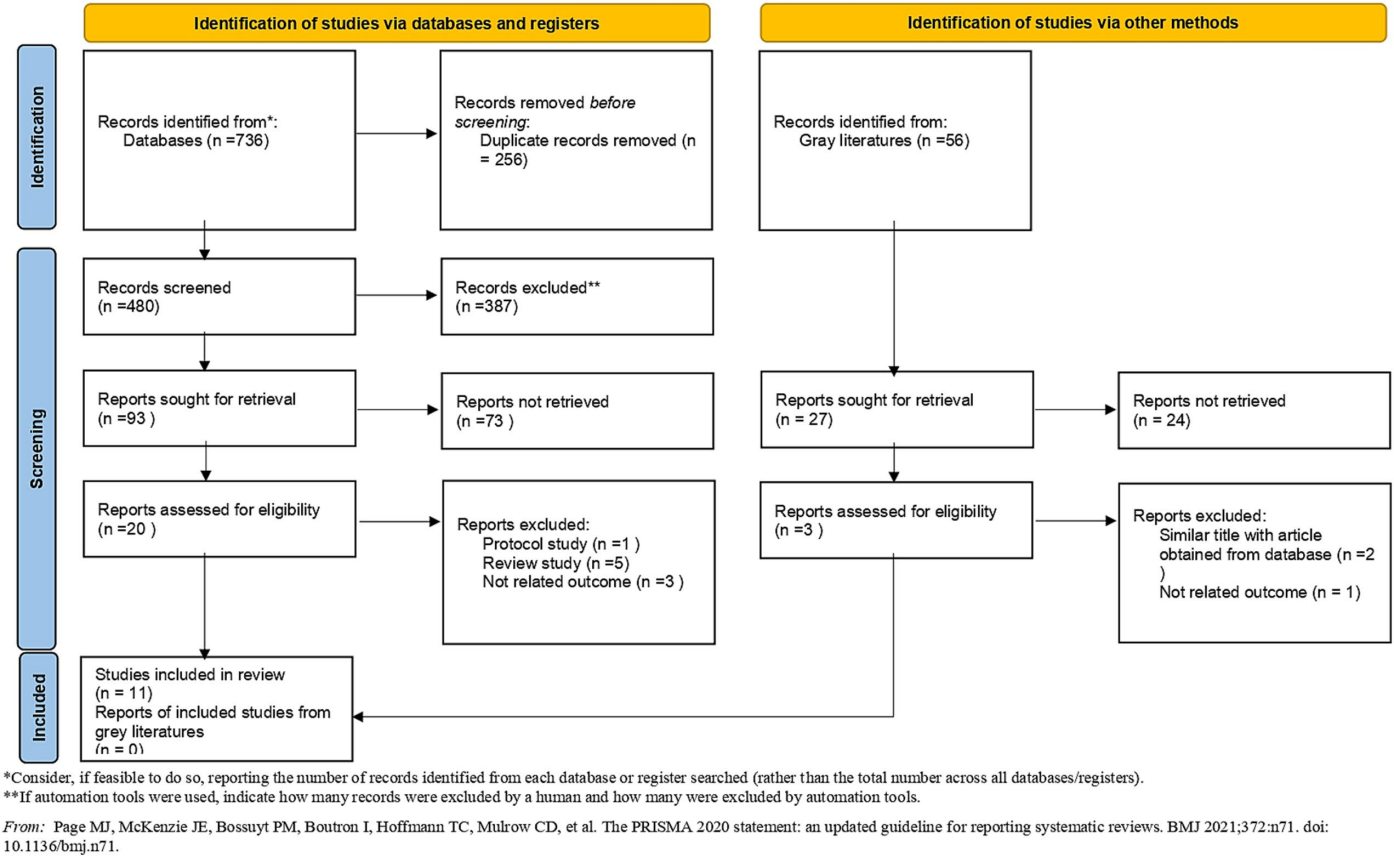
Preferred Reporting Items for Systematic Reviews and Meta-Analyses flow diagram (2020) of search process.

The comprehensive search results and the methodology used for identification are presented in the PRISMA flow diagram (Figure 1). By eliminating duplicate references from our initial records, 480 studies were evaluated in the second phase of screening and finally 12 studies met the necessary criteria to include in this systematic review (23–33). Among the studies conducted, three studies were conducted on the European population and the rest on the Chinese population (Table 1). The region of amplification of the 16S rRNA gene varied across studies, such as V1, V2, V3 and V4. In all studies, the 16S rRNA gene was amplified. In the studies reviewed, the following sequencing platforms were utilized: three studies used the Illumina MiSeq platform, two studies used the Illumina HiSeq platform, one study used the Ion Torrent PGM platform, one study used the Illumina NovaSeq 6000 platform and three studies employed Mendelian randomization analysis that the type of platform used in the sequencing was not specified in some studies. In the Mendelian randomization analyses, the MiBioGen International Consortium, FinnGen, and the Dutch Microbiome Project databases were utilized to investigate causal relationships. In other studies, SILVA, KEGG, NCBI, Gold, UCLUST, HMDB, Ribosomal Database Project, RDP, and KO databases were used.

**Table 1:** Baseline characteristics of included studies.

Study ID	Study design	Population ancestry	Sample size/control	Stool sample collection method	DNA extraction method	Region amplified	Database used	Sequencing platform	Microbiome change
Jiang 2024 (25)	Mendelian randomization Analysis	European	810/391613	NR	NR	16S ribosomal RNA gene	MiBioGen International Consortium FinnGen	NR	*Coprococcus3 and Gordonibacter had a risk effect on ITP* *Methanobacteria, BacteroidalesS24.7 group,* *Lachnospiraceae, Methanobacteriaceae, Eubacterium hallii group, Eubacterium ruminantium group, Allisonella, Coprococcus2, Bacillales, Methanobacteriales are negatively associated with ITP risk*
Li 2024 (26)	Large-scale bidirectional Mendelian randomization study	European	882/405762	NR	NR	16S rRNA gene (V4, V3-V4, and V1-V2)	MiBioGen International Consortium Dutch Microbiome Project FinnGen R10	NR	*Alcaligenaceae, Escherichia Shigella* and *Odoribacter splanchnicus* associated with ITP as risk factors *Bacteroidales S24 7group, Allisonella, Coprococcus2, Eubacterium hallii, Eubacterium ruminantium, Dialister invisus, Bilophila unclassified, Haemophilus parainfluenzae, Bacteroides finegoldii* and *Dorea unclassified* were associated with ITP as protective factors
Guo 2023 (24)	Mendelian randomization study of two samples	European	703/337408	NR	NR	16S rRNA	MiBioGen International Consortium FinnGen	NR	*Alcaligenaceae had a risk effect on ITP* *Methanobacteriaceae is negatively associated with ITP risk*
Xiangyu 2023 (27)	Case-control	Chinese	25/60	Fresh stool sample collected with single-use sterility kit and refrigerated at −80°C	E.Z.N.A.® soil DNA Kit (Omega Bio-tek, Norcross, GA, USA)	16S (V3–V4) primer pairs 338F (5′-ACTCCTACGGGAGGCAGCAG-3′) and 806R (5′-GGACTACHVGGGTWTCTAAT-3′)	NR	MiSeq platform (Illumina, San Diego, CA, United States)	*Bacteroidetes, Bacteroides P1, Bacteroidetes p2, Escherichia-Shigella p3 were enriched in ITP patients* *Actinobacteria, Pseudomonas and Bifidobacterium were decreased in ITPs patient*
Rui 2023 (29)	Case-control	Chinese	37/36	NR	Zymo Research BIOMICS DNA Microprep Kit	16S rRNA (V4) 5′-3′: 515F (5′-GTGYCAGCMGCGCGGTAA-3′) and 806R (5′-GGACTACHVGGGTWTCTAAT-3′)	SILVA Database KEGG Database NCBI Database Gold Database	Illumina Novaseq6000	*Firmicutes, Proteobacteria, Epsilon bacteaeota,* *Helicobacter, Peptostreptococcaceae, Clostridium* *sensu stricto 1, and Lachnospiracea UCG-008* *microbiome Helicobacter Pylori were enriched in ITP patients* *After treatment,* *Proteobacteria, Epsilon bacteraeota, Bacteroidetes* *and Firmicutes were significantly higher*
Xiaomin (32)	Case-control	Chinese	29/33	Fecal and plasma sample	DNA extraction kit (QIAGEN)	16S rDNA (V4) 515F (50-GTGCCAGCMGCCGCGGTAA-30), 806R (50GGACTACHVGGGTWTCTAAT-30).	UCLUST and SILVA Databases KEGG Databases HMDB Databases	Illumina HiSeq	*Proteobacteria, Chloroflexi, Bacteroides, Phascolarctobacterium and Lactobacillus were enriched in ITP patients* *Firmicutes, Actinobacteria, Ruminococcaceae, Eubacterium coprostanoli, Megamonas and Lachnospiraceae NC2004 were decreased.*
Zhang 2020 (33)	Case control	Chinese	30/29	NR	QIAamp Fast DNA Stool Mini Kit (Qiagen, Hilden, Germany)	16S rRNA (V3–V4)	NCBI Sequence Read Archive (SRA) database Ribosomal Database Project Silva (SSU123) HMDB database	Illumina MiSeq	*Lactobacillus and Streptococcus, Leuconostocaceae, were enriched in ITP patients* *Bacteroidetes, Bacteroides and Bacteroides vulgatus ATCC8482 were decreased*
Wang 2023 (30)	Case control	Chinese	40/33	Fresh stool sample collected and refrigerated at −80°C	E.Z.N.A.®Soil DNA Kit (Omega Bio-tek, Norcross, GA, USA)	16S rRNA (V3–V4) 338F (5′-ACTCCTACGGGAGGCAGCAG- 3′) and 806R (5′-GGACTACHVGGGTWTCTAAT-3′).	NCBI NR database KEGG databases RDP	Illumina’s Miseq PE300 platform Illumina NovaSeq	*Proteobacteria,* *Streptococcus, Hungatella (H. hathewayi), F Ruminococcaceae, Klebsiella,* *unclassified F Enterobacteriaceae, Phascolarctobacterium, Citrobacter and Streptococcus were enriched in ITP patients* *Bacteroidetes and Romboutsia (R. timonensis) were decreased.*
Wang 2021 (31)	Cohort study	Chinese	Number of cases: 99 Number of controls:52	Fresh stool sample collected and refrigerated at −80°C	QIAamp DNA Stool Mini Kit (Qiagen, Germany)	NR	NCBI Database KEGG databases KO databases	Illumina Hiseq PE150	*Pedobacter, Bifidobacterium scardovii, Clostridium tyrobutyricum,* *Prevotella* sp. *oral taxon 472 and butyrate-producing* *bacterium SM4/1 were enriched in corticosteroid resistant ITP patients* *Lachnobacterium and Rhodonellum in corticosteroid resistant ITP patients were decreased.*
Liu 2020 (28)	Case control	Chinese	94/93	Fecal samples were collected using disposable sterile kits (Taixing Wantong Medical Equipment Factory, Taixing, China) and refrigerated at −80°C	stool DNA extraction (QIAamp DNA Stool Mini Kit, Qiagen, Germantown, MD, USA)	16S rRNA (V4) 515F (AATGATACGGCGACCACCGAGATCTACACNNNNNNNNTATGGTAATTGTGTGCCAGCMGC)	NR	Ion Torrent PGM	*Actinobacteria, Bacteroidetes, Cyanobacteria/Chlorop, Members of Anaerorhabdus, Fusobacteria, and Verrucomicrobia were enriched* *Firmicutes, Proteobacteria, and Synergistetes were decreased.*

NR, Not Reported; ITP, Immune Thrombocytopenia purpura; MiBioGen, Microbiology and Bioengineering; FinnGen, Finnish Genetics; rRNA, Ribosomal RNA; BIOMICS, Biomolecule Isolation and Microbial Characterization System; SILVA Database, Sequence Identification; Linkage and Variation Analysis Database; KEGG Database, Kyoto Encyclopedia of Genes and Genomes Database; NCBI Database, National Center for Biotechnology Information.

### Phylum

Four studies indicated a significant increase in the percentage of *Bacteroidetes* in patients with ITP (27, 28, 32, 33). Conversely, two studies reported a decrease in the percentage of *Bacteroidetes* in the gut microbiota of patients with ITP (30, 33).

One study indicated that the percentage of *Firmicutes* among the patients with ITP was significantly increased (29). In contrast, two studies reported a decrease in the percentage of *Firmicutes* in the gut microbiota of ITP patients (28, 32).

In three studies, it was reported that the phylum *Proteobacteria* is increased in patients with ITP (30, 32, 33). However, one study reported a decrease in the phylum *Proteobacteria* among the patients with ITP (28).

In the context of *Actinobacteria*, two studies reported a decrease in its percentage in the gut microbiota of patients with ITP (27, 32), while one study indicated an increase in the percentage of Actinobacteria in the case group (28).

### Others phylum

*Cyanobacteria/Chloroplast, Fusobacteria, Verrucomicrobia* (28), *Chloroflexi, Phascolarctobacterium*, *Epsilonbacteaeota* (32) were increased in ITP patients, while *Synergistetesdecreased* in patients (28).

### Genus

*Gordonibacter, Coprococcus3* (25), *Bacteroides P1, Escherichia, Shigella p3* (27), *Odoribacter splanchnicus* (26), *Helicobacter, Peptostreptococcaceae, Clostridium sensu stricto 1, Lachnospiracea UCG-008* (29), *Phascolarctobacterium* (30, 32), *Lactobacillus* (32, 33), *Streptococcus* (30, 33), *Weissella* (33), *Hungatella, Klebsiella*, *Citrobacter, Enterobacteriaceae* (30), *Bifidobacterium, Pedobacter* (31), and *Anaerorhabdus* (28) are increased in genus level.

*Eubacterium ruminantium, Allisonella, Eubacteriumhallii, Coprococcus2* (25, 26), *Pseudomonas, Bifidobacterium* (27), *Ruminococcaceae, Megamonas, Romboutsia* (32), *Haemophilus parainfluenzae, Bacteroides finegoldii, Dorea* (26), *Lachnobacterium* and *Rhodonellum* (31) decrease in genus level.

In the genus level, both *Bifidobacterium* and *Lachnobacterium* have been reported to show both increases and decreases in patients with ITP. This variability suggests that the gut microbiome’s composition can differ significantly among individuals with ITP, indicating a complex relationship between these genera and the disease.

### Alpha and beta diversity

The results of alpha and beta diversity evaluation in ITP patients are summarized in Table 2. As shown in Table 2, seven studies reported results about alpha and beta diversity. Most of the studies used the Shannon index and PCoA methods for alpha and beta diversity, respectively. Among the evaluated studies three studies found a significant reduction in alpha diversity in patients with ITP (26, 29, 32). However, in three studies (28, 31, 33), there wasn’t any difference among the ITP group and control group in term of alpha diversity and in one study (30) patients with ITP showed a significant increase in alpha diversity compare than control group.

**Table 2:** The results of alpha and beta diversity among the ITP patients.

Study ID	Alpha diversity method	Alpha diversity and richness	Beta diversity method	Beta diversity
Li 2023 (27)	Chao 1, Shannon indices	Lower Shannon index in the ITP group	PCoA	PCoA analysis indicated that the overall structure of Gut microbiota of the ITP group was significantly different from that of the control group
Rui 2023 (29)	Chao1, ACE, Shannon, Simpson, Pielou, Indices	Lower Shannon index in the ITP group	Bray-Curtis distance, PCoA UniFrac Method	Improper overall composition of the gut microbiota in the ITP group
Yu 2022 (32)	Chao1, phylogenetic diversity (PD), Simpson and Shannon	All of the methods showed a significant reduction in the microbiome diversity among the ITP patients	PCoA	Improper significant changes in case group
Zhang 2020 (33)	Chao 1 and Shannon Indices	No significant differences among tow group	PCoA	Significant differences among tow group
Wang 2023 (30)	Alpha diversity of the ITP group was higher than that of healthy controls	Higher Sobs and Shannon indexes, and lower Simpson index	PCoA and NMDS	The intestinal microbiome of ITP patients is disorganized
Wang 2021 (31)	Shannon index	No significant differences among groups	Bray-Curtis distance	β-diversity in ITP gut microbiome was higher than that in controls at both the gene level and the species level
Liu 2020 (28)	Chao, Ace, Sobs, Shannon and Simpson indices	No significant differences between ITP groups and control groups	UniFrac distances by PCoA	Overall structures of the gut microbiota of the ITP and control groups were significantly different

ITP, Immune Thrombocytopenia purpura; PCoA, Posterior Communicating Artery; ACE, Automated Customer Experience; UniFrac, unique fraction; NMDS, National Minimum Data Set; Chao, Chief Accounting Officer; Sobs, Standard Operating Procedures; Shannon, Shannon Information Theory.

## Discussion

### Key microbiota changes in ITP

The role of gut microbiota in the immune system has become a popular topic in recent years (34). The gut microbiota plays a crucial role in immune system balance from birth (35). This systematic review explored the alterations in gut microbiota composition in patients with ITP.

At the phylum level, the most commonly reported changes in ITP patients involved Firmicutes, Bacteroidetes, Proteobacteria, and Actinobacteria. Several studies reported an increase in Bacteroidetes (27–29, 32), whereas others found a decrease (30, 33). Similarly, Firmicutes exhibited inconsistent findings, with both increases (29) and decreases (28, 32) reported. Proteobacteria levels were elevated in some studies (30, 32, 33), while others reported a decline (28). Actinobacteria also showed inconsistent trends, with increases (28) and decreases (27, 32) in different studies. These discrepancies may be due to variations in study populations, methodologies, or disease heterogeneity.

These findings suggest that microbial dysbiosis plays a role in ITP pathogenesis, though inconsistencies exist across studies, potentially due to population differences, dietary habits, or sample size variations (32). Additionally, differences in sequencing platforms used across studies may contribute to variations in microbial composition results. Platforms such as Illumina MiSeq, HiSeq, NovaSeq, and Ion Torrent PGM utilize different sequencing depths, read lengths, and bioinformatics pipelines, which can affect taxonomic classification and microbial diversity assessments (36). Moreover, ITP itself is a heterogeneous condition, and variations in results may also be influenced by the phase and type of ITP (primary vs. secondary) as well as the impact of different therapies. Corticosteroids, TPO-RAs, and immunosuppressive agents can alter gut microbiota composition, potentially affecting study outcomes (28).

### Mechanisms linking dysbiosis to disease development

Gut microbiota plays a crucial role in maintaining immune homeostasis, metabolic function, and barrier integrity. Dysbiosis, or microbial imbalance, can contribute to various diseases through several mechanisms (37). Immune dysregulation occurs when changes in microbial composition lead to an overactive immune response, contributing to autoimmune diseases such as rheumatoid arthritis, multiple sclerosis, and ITP (38). Certain bacteria promote Th17 cell activation, which is linked to autoimmune inflammation, while others support regulatory T cells (Tregs) that suppress excessive immune activation. Another mechanism involves altered metabolite production, where beneficial bacteria produce short-chain fatty acids (SCFAs) (e.g., acetate, butyrate, and propionate), which have anti-inflammatory effects and maintain gut barrier integrity (36). Dysbiosis reduces SCFA levels, leading to increased gut permeability (“leaky gut”) and systemic inflammation. Additionally, the accumulation of harmful metabolites like trimethylamine N-oxide (TMAO) may contribute to platelet activation and cardiovascular risks (33). Molecular mimicry and autoimmunity is another pathway, where microbial antigens resemble host proteins, leading to cross-reactive immune responses that can trigger autoimmunity (32). This mechanism has been implicated in diseases such as lupus, type 1 diabetes, and potentially ITP. Furthermore, the gut-brain and gut-platelet axis links the gut microbiota to the nervous and hematologic systems through metabolites and immune signaling (27).

### Metabolites of interest in ITP

Gut microbiota influences host immunity through microbial metabolites. In ITP, SCFAs, polyamines, and TMAO appear to play a significant role. There is a relationship between platelet count and the gut microbiota. Some gut microbiota reduce platelet counts by decreasing inflammatory factors such as interleukin-6 and CRP (39). Metabolites produced by the gut microbiota have a strong effect on platelet function. One of the metabolites produced by the human gut microbiota is sialic acid, which has an inhibitory effect on megakaryocytes in the bone marrow (40). The gut microbiota, by producing trimethylamine N-oxide, leads to increased platelet activation and thrombotic conditions. In addition, SCFAs produced by *Bacteroidetes* are utilized in mitochondrial beta-oxidation for energy production and have anti-inflammatory effects. A decrease in *Bacteroidetes* is associated with the onset of autoimmune diseases (30). SCFAs are the main products resulting from the fermentation of dietary fiber by the gut microbiota and they include acetate, propionate, and butyrate. SCFAs are known to enhance the activity of type 3 innate lymphoid cells (ILC3) while suppressing the activity of type 2 innate lymphoid cells (ILC2). SCFAs modulate the immune system by: Decreasing Th17 cells, increasing regulatory CD4+ T cells (Tregs) and increases regulatory B cells and interleukin-10 production. These compounds are produced by specific subgroups of the *Bacteroidetes phylum* and the *Firmicutes phylum*. *Bacteroidetes* primarily produce acetate and propionate, while *Firmicutes* mainly produce butyrate (41).

Polyamines are organic compounds involved in immune modulation. Their levels are generally reduced in autoimmune diseases, including systemic lupus erythematosus and Hashimoto’s thyroiditis (42). However, in ITP, polyamine levels were found to be increased (43, 44). This paradox suggests that microbiota-driven polyamine synthesis may differ in ITP compared to other autoimmune disorders.

Another key metabolite, TMAO, is associated with platelet activation and thrombosis risk (40). The gut microbiota produces TMAO through the metabolism of dietary choline, and its elevation has been linked to increased platelet aggregation. This suggests a potential mechanistic link between gut dysbiosis and platelet destruction in ITP (31).

### Implications of microbiota alterations in ITP

Microbiota dysbiosis in ITP may have diagnostic and therapeutic implications. The Firmicutes/Bacteroidetes (F/B) ratio, frequently used as a marker of dysbiosis, showed conflicting results in ITP studies. Some reported an increased F/B ratio (33), while others observed a decrease (32). Given these inconsistencies, the F/B ratio alone may not be a reliable biomarker for ITP.

The function of follicular helper T (Tfh) cells, which leads to the activation of autoreactive B cells (45), along with increased activation and apoptosis of platelets dependent on CD8+ cells, is another immune mechanism contributing to platelet reduction in this disease (46). The number of megakaryocytes in the bone marrow of ITP patients is normal. Some studies indicated that ITP patients show both increased and decreased apoptosis of megakaryocytes. This suggests that different mechanisms may be involved in different patients, contributing to the heterogeneity of the disease (47). Some specific microbiomes, such as *filamentous bacteria*, *Citrobacter rodentium*, and *Escherichia coli O157*, initiate a response mediated by Th17 cells. Th17 cells and their inflammatory cytokines play an important role in the immunopathogenesis of ITP patients (48, 49). In mic models, it has been shown that the *Firmicutes phylum* can reduce colonic inflammation mediated by a decrease in Th17 cells (28). By reviewing the studies, it was found that *Firmicutes* decreased in 2 studies in ITP patients (28, 32) and increased in 1 study (29). The reason for this discrepancy could be the sample size of patients in the studies.

Several studies highlighted an association between microbiota alterations and immune mechanisms in ITP. Specific taxa, such as *Helicobacter pylori*, were linked to treatment response, with eradication leading to improved platelet counts (29). Additionally, bacterial species associated with Th17 activation (e.g., *Citrobacter rodentium* and *Escherichia coli* O157) were enriched in ITP patients (48, 49). These findings suggest a potential role for microbiota-targeted therapies in ITP management.

### Pharmacological treatments and their impact on gut microbiota

Pharmacological treatments used in ITP management can significantly alter the gut microbiota, potentially influencing disease progression and treatment response. Corticosteroids, which are commonly used as first-line therapy, have been shown to reduce microbial diversity and increase the abundance of opportunistic pathogens, while decreasing beneficial bacteria such as Lactobacillus and Bifidobacterium (50). These changes can lead to gut barrier dysfunction and immune dysregulation, which may contribute to treatment resistance or disease relapse (29).

Thrombopoietin receptor agonists (TPO-RAs), such as eltrombopag and romiplostim, have been associated with alterations in gut microbiota composition, though their effects remain less well-studied. Some evidence suggests that TPO-RAs may modulate gut bacteria through indirect effects on immune signaling and platelet function, potentially influencing inflammation and autoimmunity (31).

Immunosuppressive agents, including rituximab, azathioprine, and cyclosporine, can also disrupt gut microbiota by reducing microbial diversity and shifting the balance of immune-regulating bacteria. Rituximab, a monoclonal antibody targeting CD20, depletes B cells and may indirectly influence the gut microbiota by altering immune homeostasis. Studies have shown that B-cell depletion can lead to dysbiosis, with reductions in Clostridia and Firmicutes species that are important for maintaining gut immune balance. Antibiotic exposure, either directly or as part of ITP management, can cause significant disruptions in gut microbiota, leading to loss of beneficial bacteria and overgrowth of opportunistic pathogens. This imbalance may further exacerbate immune dysregulation and impact platelet homeostasis (51–53).

Given these microbiota-altering effects, leveraging microbiome data could help guide ITP therapy. For example, microbiota profiling before treatment initiation may help predict corticosteroid responsiveness, as patients with lower microbial diversity and increased pro-inflammatory bacteria may be at higher risk of treatment failure (54–57). Additionally, microbiome-targeted interventions such as probiotics, prebiotics, and dietary modifications could be explored as adjunct therapies to mitigate treatment-induced dysbiosis and improve treatment outcomes (33).

### Microbiota differences in responders vs. non-responders to therapy

Pharmacological treatments can alter the gut microbiota. This change is associated with alterations in the metabolites of the gut microbiota, which modify autoimmune responses (50). It was found that the levels of *Helicobacter pylori* increase in patients who respond positively to treatment (29). *H. pylori* cause a decrease in the inhibitory FcγRIIb receptor on the surface of monocytes, and by eradicating *H. pylori*, platelet counts normalize (31). Multiple studies have reported that infection with *H. pylori* is associated with decreased platelet count and ITP (51–53). It should be noted that the sample size of this study was small (37 participants) and that the gut microbiome was assessed 4 weeks after treatment. In addition, *H. pylori* is often a pathogenic agent. Given the small sample size and the results of previous studies, the findings of this study cannot be trusted. The *Firmicutes/Bacteroidetes* ratio is used to assess gut microbiome dysbiosis (54–57). In this study, this ratio did not show a significant difference between the treated patient group and healthy individuals. Based on the search results, the *Firmicutes/Bacteroidetes* ratio has shown inconsistent results across different studies: one study, this ratio increased (33) and another one this ratio was decrease (32). Based on the heterogeneity of the results across the studies, it cannot be concluded that the F/B ratio is a reliable diagnostic finding or a marker for monitoring treatment in ITP patients.

Patients responsive to corticosteroid therapy exhibited distinct microbiota profiles compared to non-responders. Notably, Lachnobacterium and Rhodonellum were decreased in corticosteroid-resistant patients, while Pedobacter was increased (31). Moreover, analysis of glycosaminoglycans and dermatan sulfate degradation pathways revealed metabolic differences between responders and non-responders, suggesting potential microbiota-driven mechanisms underlying corticosteroid resistance.

Geographical differences in gut microbiota composition have been well documented in various diseases, including autoimmune disorders (58). The studies included in this review predominantly assessed two major populations: European and Chinese. While the core microbiota remains largely conserved, notable regional variations exist due to genetic, dietary, and environmental factors (59). European studies identified increased relative abundance of Coprococcus3 and Gordonibacter as risk factors for ITP, whereas Chinese studies highlighted an increase in Escherichia-Shigella and *Odoribacter splanchnicus* (60). Additionally, the Firmicutes/Bacteroidetes ratio showed inconsistent trends between the two populations, potentially reflecting differences in diet and antibiotic exposure. The variations in microbiota composition suggest that distinct mechanisms may contribute to ITP pathogenesis in different populations, underscoring the need for region-specific microbiome analyses in future studies (61).

This systematic review adheres to the highest standards of evidence synthesis, following the Cochrane Handbook and PRISMA 2020 guidelines. The search strategy was peer-reviewed, and multiple databases were exhaustively searched, including both published and unpublished studies, ensuring a comprehensive assessment of available evidence. Also, this review addresses a critical gap in understanding the role of the gut microbiome in the pathophysiology of ITP, offering new insights into microbial dysbiosis and its immunological impact on platelet function. However, the present study had some limitation that should be considered. First, the studies included in this review exhibit high methodological heterogeneity in terms of study populations, sequencing platforms, and analytical techniques. Second, the majority of the studies reviewed were conducted on Chinese and European populations. This limited geographic representation may hinder the generalizability of the findings to other populations, particularly in regions with differing dietary habits, environmental factors, and genetic backgrounds. Third, Several of the studies included in this review had small sample sizes, which may introduce bias and limit the statistical power of their findings.

## Conclusion

This systematic review highlights the emerging role of the gut microbiome in the pathophysiology of ITP, shedding light on potential microbial dysbiosis that may influence platelet regulation and immune dysfunction. The findings suggest alterations in specific microbial taxa and a disruption in microbial diversity, which could contribute to immune-mediated platelet destruction or impaired thrombopoiesis. However, the heterogeneity of existing studies, small sample sizes, and lack of longitudinal data underscore the need for more robust, well-designed research to establish causal relationships and identify therapeutic microbial targets. Future investigations incorporating diverse populations and standardized methodologies are essential to fully elucidate the gut microbiome’s potential role in the onset, progression, and treatment of ITP. Understanding this complex interplay could open new avenues for microbiome-based interventions, offering a novel approach to the management of ITP.

## Data Availability

The raw data supporting the conclusions of this article will be made available by the authors, without undue reservation.
